# Clinical Characteristics and Survival Outcomes for Non-Small-Cell Lung Cancer Patients with Epidermal Growth Factor Receptor Double Mutations

**DOI:** 10.1155/2018/7181368

**Published:** 2018-01-16

**Authors:** Min Peng, Yi Ming Weng, Hua Li Liu, Gui Fang Yang, Yi Yao, Guang Han, Qi Bin Song

**Affiliations:** ^1^Department of Oncology, Renmin Hospital of Wuhan University, Wuhan University, Wuhan, Hubei 430060, China; ^2^Department of Pathology, Zhongnan Hospital of Wuhan University, Wuhan University, Wuhan, Hubei 430060, China; ^3^Department of Oncology, Hubei Cancer Hospital, Wuhan, Hubei 430060, China

## Abstract

Multiple randomized clinical trials have demonstrated that epidermal growth factor receptor (EGFR) exon 19 deletion (19Del) and exon 21 L858R mutation (L858R) are highly correlated with sensitivity to epidermal growth factor receptor tyrosine kinase inhibitor (EGFR-TKI) treatment in non-small-cell lung cancer (NSCLC). A mutation in exon 20 (T790M) is reportedly associated with resistance to EGFR-TKIs. However, few studies have focused on patients harboring double mutations in these 3 mutation sites. In this retrospective study, forty-five patients (45/2546, 1.7%) harbored double mutations of 19Del, L858R, and T790M. Twenty-four patients with EGFR double mutations received EGFR-TKI therapy. Clinical characteristics of these patients, including the response to EGFR-TKIs and progression-free survival outcome for EGFR-TKI treatment (PFS-TKI), were analyzed. Patients with EGFR double mutations were more likely to be nonsmokers, have an Eastern Cooperative Oncology Group Performance Status (ECOG PS) of 0-1, have adenocarcinoma, and be at stage III-IV. The ORR, DCR, and median PFS-TKI in patients harboring EGFR double mutations were lower than in patients with a single EGFR-activating mutation. The differences in ORR and DCR were statistically insignificant between the 3 groups. Patients with double mutations of 19Del and T790M had longer PFS-TKIs than patients in the other 2 groups.

## 1. Introduction

Lung cancer has the highest incidence of all cancers and is the leading cause of cancer-related death worldwide. Non-small-cell lung cancer (NSCLC) accounts for approximately 80% of lung cancer cases [1]. Epidermal growth factor receptor (EGFR) is a transmembrane glycoprotein which is overexpressed in more than 40% of NSCLC. Mutations of EGFR gene are present in approximately 50% of NSCLC patients in Asia [2]. The 2 most common EGFR mutations have been identified as exon 19 deletion (19Del) and exon 21 point mutation (L858R) and account for 85–90% of EGFR mutations in NSCLC. In addition, 19Del and L858R are highly associated with sensitivity to EGFR tyrosine kinase inhibitors (EGFR-TKIs) [3]. Patients with either of these 2 mutations experience dramatic improvements in survival, symptoms, and quality of life from EGFR-TKI treatment compared with traditional platinum-based chemotherapy [4].

Occasionally, 19Del mutation and L858R mutation are present together in a single tumor sample. The response to EGFR-TKIs in NSCLC patients with this double mutations is unclear. Currently, the presence of this double mutations is not so rare that we need know the efficacy of EGFR-TKIs for patients with such mutations [5–7]. However, few reports have described the clinical characteristics and response to EGFR-TKIs of NSCLC patients with concomitant mutations of 19Del and L858R, and case numbers have been limited [3, 8, 9].

Despite sensitivity to EGFR-TKIs, the majority of NSCLC patients with activating mutations acquire TKI resistance after a median of approximately 10 months from the onset of EGFR-TKI therapy. Although multiple mechanisms are reported, a second mutation of threonine-to-methionine substitution at amino acid position 790 (T790M) in exon 20 accounts for more than half of the acquired resistance to EGFR-TKIs [10]. Recently, studies have reported that T790M might coexist with EGFR-activating mutations in the same cancer sample before EGFR-TKI treatment, despite the low incidence. A meta-analysis demonstrated that, prior to treatment, EGFR T790M mutation was more likely to coexist with L858R mutation than with exon 19 deletion in NSCLC [11]. Moreover, clinical studies have implied that concomitant mutations of T790M and activating mutations might represent resistance to TKIs [12–15]. However, some case reports describe NSCLC patients with these double mutations responding well to EGFR-TKIs [16–18]. Researchers also observed that the growth rate was lower in cell lines with double mutations of 19Del and T790M than in cell lines with 19Del mutation alone [19, 20]. Owing to the very low incidence and small sample size, the clinical significance of these double mutations has never been systematically analyzed.

The purposes of our study were to explore the prevalence of EGFR double mutations in NSCLC patients and their clinical characteristics before EGFR-TKI treatment. In addition, we also analyzed the response to EGFR-TKIs and compared the progression-free survival outcomes for EGFR-TKI treatment (PFS-TKIs) between NSCLC patients with different EGFR double mutation types.

## 2. Materials and Methods

### 2.1. Patients and Data Collection

Inclusion criteria were as follows: (1) patients with pathologically confirmed NSCLC who underwent EGFR mutation screening and treatment at Renmin and Zhongnan Hospitals of Wuhan University and Hubei Cancer Hospital between March 2007 and November 2016; (2) patients who underwent complete cancer staging, including chest computed tomography (CT) scan, abdominal ultrasound/CT, bone scan, and MRI of the brain, prior to treatment; (3) clinical stage was classified using the tumor, node, metastasis (TNM) system proposed by the American Joint Committee on Cancer (7th edition); and (4) patients who harbored double mutations 19Del + L858R, 19Del + T790M, or L858R + T790M. A total of 45 patients were enrolled into the study. Clinical characteristics, including age, gender, smoking history, clinical stage, pathological type, Eastern Cooperative Oncology Group (ECOG) performance status (PS), type of TKI, response to EGFR-TKIs, and PFS-TKI, were reviewed from medical records. Patients with unknown treatment histories were excluded from therapeutic analysis. Response was classified by standard Response Evaluation Criteria in Solid Tumors (RECIST). PFS-TKI was defined as the time from the first day of EGFR-TKI therapy until radiological progression or death. Patients lost to follow-up were censored at the date of last contact. The study protocol was reviewed and approved by the Review Board and Ethics Committee of Renmin and Zhongnan Hospitals of Wuhan University and Hubei Cancer Hospital, and the research was carried out in accordance with approved guidelines and the Declaration of Helsinki. All patients provided written informed consent before any study-related procedure.

### 2.2. EGFR Mutation Testing

Tumor specimens, including paraffin blocks or frozen tissues of surgical specimens, were used to test EGFR mutation. Tumor tissue was scraped from glass slides under direct visualization or under a dissecting microscope. DNA was derived from tumors embedded in paraffin blocks using a QIAmp DNA Mini kit (Qiagen). EGFR mutations were detected using the YUANQI EGFR Mutations Detection Kit (YUANQI Bioscience Co. Ltd., Shanghai, China). The assay was carried out using ViiA7 DX real-time fluorescent quantitative PCR (Applied Biosystems, Foster City, USA), according to manufacturer's protocol.

### 2.3. Statistical Analysis

All analyses were performed using SPSS for Windows Version 20.0 (IBM, Chicago, USA). Clinical characteristics of the different groups, including age, gender, smoking history, clinical stage, pathological type, ECOG PS, and response to EGFR-TKI treatment, were compared by Fisher exact test. The PFS-TKIs were calculated using the Kaplan–Meier method. The survival curves between different groups were compared using the log-rank test. The statistically significant variables in the log-rank test were evaluated by Cox regression. All statistical tests were two-sided, with significance defined as *P* < 0.05.

## 3. Results

### 3.1. Baseline Patient Characteristics

A total of 2546 patients were consecutively enrolled into the study at initial diagnosis. The incidence of EGFR single mutation and double mutations among detected NSCLC patients were 48.1% (1225/2546) and 2.4% (61/2546), respectively. 576 patients were confirmed as 19Del mutation. 550 patients hold L858R mutation. Fourteen (0.55%) patients had double mutations of 19Del + T790M. Seventeen (0.67%) and fourteen (0.55%) patients were identified as having concomitant mutations of 19Del + L858R and L858R + T790M, respectively. The remaining 16 (0.63%) patients had EGFR double mutations containing rare EGFR mutation types, including G719X, S768I, and L861Q. Because the number of patients in each rare EGFR mutation group was small (L858R + 20Ins, *n* = 2; G719X + S768I, *n* = 4; S768I + L858R, *n* = 6; G719X + T790M, *n* = 1; G719X + L861Q, *n* = 1; 19Del + L861Q, *n* = 1; G719X + 19Del, *n* = 1), we excluded them from further analysis.

Of the 45 patients with common EGFR double mutations, 23 were female. Patient age ranged from 39 to 76 years with a median age of 58 years. Most of the patients were stage III-IV (*n* = 32, 71.1%). Thirty-two (71.1%) patients were never smokers. Histology revealed adenocarcinoma in 43 (95.6%) patients and squamous cell carcinoma in 2 (4.4%) patients. Characteristics of those patients with common EGFR double mutations, including age, gender, smoking history, clinical stage, pathological type, ECOG PS, and the use of EGFR-TKIs, are summarized in Table 1.

Fourteen of 45 patients (31.1%) showed concomitant mutations 19Del + T790M. In this group, 5 patients were male and only 2 patients were smokers or former smokers. Only 1 patient was identified with squamous cell carcinoma. The distributions of patients in stage I-II and III-IV were 6 and 8, respectively. Fourteen of 45 patients (31.1%) were identified with the double mutation of L858R + T790M. This group comprised 4 men and 10 women. Four patients were smokers or former smokers and 10 patients were never smokers. Most of the patients were stage III-IV disease (10 patients, 71.4%). Finally, 17 (37.8%) patients had the double mutation of 19Del + L858R, including 16 patients with adenocarcinoma and 1 patient with squamous cell carcinoma. Results indicated that patients in this group were more likely to be male (13 patients, 76.5%), never smokers (10 patients, 58.8%), and have stage III-IV disease (14 patients, 82.4%). Compared with the other 2 groups, patients with EGFR double mutations of 19Del + L858R were more likely to be male (76.5% versus 35.7% versus 28.6%, *P* = 0.0145; Table 1). No significant difference was observed between the 3 groups in terms of other baseline characteristics, including age, smoking history, clinical stage, PS, pathological type, and use of EGFR-TKIs.

The above baseline characteristics had also been compared between each subgroup and patients with single common EGFR mutation (19Del or L858R mutation). Compared with single common mutation group, there was a higher proportion of male (76.5% versus 41.3%, *P* = 0.0035; Table S1) in 19Del + L858R group. In addition, in 19Del + T790M group, more patients were in clinical stage I-II (42.9% versus 14.8%, *P* = 0.0037; Table S2). However, there was no significant difference between L858R + T790M group and single common mutation group (Table S3).

### 3.2. Efficacy of EGFR-TKIs

Among the 45 patients with EGFR double mutations, 24 patients received EGFR-TKI therapy, including 10 patients as first-line therapy, 12 patients as second-line therapy, and 2 patients as third-line therapy. 12, 9, and 3 patients received gefitinib, icotinib, and erlotinib treatment, respectively. All the patients who received EGFR-TKI treatment were III-IV stage or had recurring disease. The objective response rate (ORR) and disease control rate (DCR) were 25% (6/24) and 62.5% (15/24), respectively. No patient had a complete response (CR) from EGFR-TKI therapy.

Table 2 shows the baseline characteristics of those patients who received EGFR-TKI therapy. Compared with the other 2 groups, patients in the 19Del + L858R group were more likely to be male (81.8% versus 37.5% versus 20%, *P* = 0.0363) and had a higher rate of smoking (63.6% versus 0% versus 20%, *P* = 0.0114). No difference was observed between the 3 groups in terms of other clinical characteristics, such as age, EGFR-TKI usage, pathological type, and PS.

In 17 patients with EGFR double mutation of 19Del + L858R, 11 (64.7%) patients underwent EGFR-TKI therapy. The ORR and DCR were 18.2% (2/11) and 45.5% (5/11), respectively. Six and five patients received EGFR-TKI as first-line and second-line therapy, respectively. Five patients were treated with gefitinib and six patients received icotinib. In 14 patients with concomitant mutations of L858R + T790M, only 5 (35.7%) patients had EGFR-TKI therapy, including 2 patients as first-line and 3 patients as second-line therapy. Three and two patients used gefitinib and icotinib, respectively. Four patients were identified as stable disease (SD) and 1 patient failed to respond to EGFR-TKIs. The ORR and DCR of these 5 patients were 0% (0/5) and 80% (4/5), respectively. There were 14 patients with double mutations of 19Del and T790M. In this group, 8 patients received EGFR-TKI therapy, including 2 patients as first-line therapy, 4 patients as second-line therapy, and 2 patients as third-line therapy. 4, 1, and 3 patients received gefitinib, icotinib, and erlotinib treatment, respectively. The ORR and DCR for the 8 patients who received EGFR-TKIs were 50% (4/8) and 75% (6/8), respectively.

Although patients with double mutation 19Del + T790M had the highest ORR (50%), the differences in ORR were statistically insignificant compared with patients with concomitant mutations of 19Del + L858R (50% versus 18.2%, *P* = 0.141) or L858R + T790M mutations (50% versus 0%, *P* = 0.057). The differences in DCR were also not obvious (*P* = 0.2794) between the 3 groups. Fisher exact test was employed to assess the correlation of clinical factors with ORR and DCR. None of those factors (age, gender, smoking history, EGFR-TKI line, pathological type, PS, and type of EGFR-TKI) were confirmed to be related to ORR or DCR.

### 3.3. Progression-Free Survival Outcomes for EGFR-TKI Treatment

Twenty-four patients with EGFR double mutations received EGFR-TKI therapy and the median PFS-TKI was 5.95 months (ranging from 0.5 to 38.2 months). In 11 patients with double mutations of 19Del + L858R who received EGFR-TKI treatment, 10 patients had disease progression and 1 patient still responded to EGFR-TKI. The median PFS-TKI was 3.3 months (ranging from 0.5 to 20.2 months). Among the 5 patients with concomitant mutations of L858R + T790M who underwent EGFR-TKI treatment, 4 patients were identified with radiological progression. The median PFS-TKI of those patients was 3.0 months (ranging from 0.9 to 9.8 months). Of the 8 patients with double mutation 19Del + T790M who were treated with EGFR-TKIs, 7 patients had disease progression. The median PFS-TKI was 16.5 months (ranging from 1.1 to 38.2 months).

**Figure 1:**
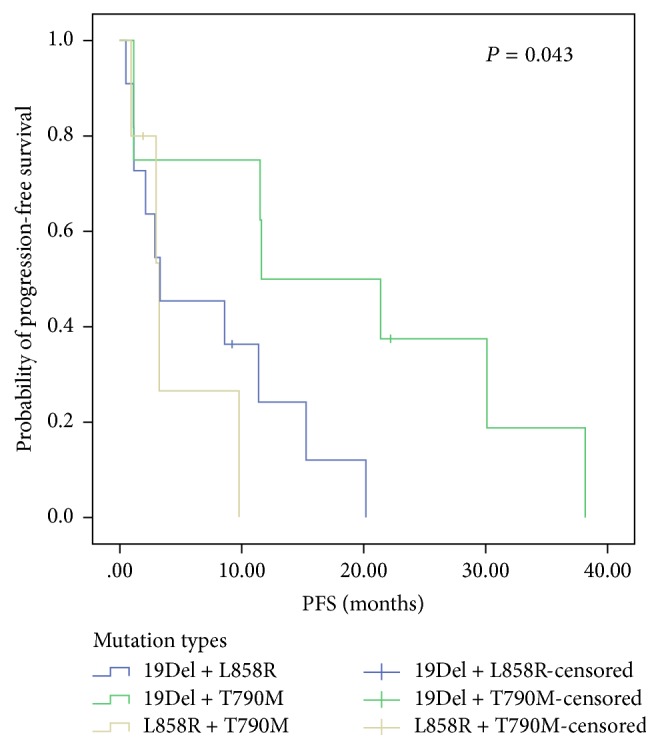
Kaplan–Meier curve for PFS of patients with different EGFR double mutation types treated with EGFR-TKIs.

We used the log-rank test to analyze the correlations between clinical characteristics (age, gender, smoking history, PS, EGFR-TKI line, pathological type, response to EGFR-TKIs, type of EGFR-TKI, and genotype) and PFS-TKIs. The results showed that patients who achieved a partial response (PR) from EGFR-TKIs had a better PFS-TKI than those with SD (*P* = 0.040). Interestingly, we found that patients with double mutations of 19Del and T790M had greater improvements in PFS-TKI than the other 2 groups (19Del + L858R versus 19Del + T790M versus L858R + T790M, 3.3 m versus 16.5 m versus 3.0 m, *P* = 0.043; 19Del + T790M versus L858R + T790M, 16.5 m versus 3.0 m, *P* = 0.047; 19Del + L858R versus 19Del + T790M, 3.3 m versus 16.5 m, *P* = 0.036; 19Del + L858R versus L858R + T790M, 3.3 m versus 3.0 m, *P* = 0.473) (Figure 1). No significant association was identified between PFS-TKI and other clinical factors. Multivariate Cox regression showed that patients with the double mutations of 19Del + T790M were associated with longer PFS-TKI than L858R + T790M (Table 3). Because of a high censoring rate of 75.6% (34/45), overall survival (OS) data are still immature and will be reported after additional follow-up.

## 4. Discussion

Many studies have concerned the clinical characteristics and survival outcomes of NSCLC patients with single common EGFR mutations such as 19Del, L858R, and T79 M [21–25]. 19Del and L858R, which constitute approximately 50–90% of total EGFR mutations, have been repeatedly confirmed as sensitive mutations for EGFR-TKIs. Somatic EGFR T790M mutation is known to occur as a “secondary mutation” in more than 50% of patients who acquire resistance to EGFR-TKIs [26]. However, few trials have investigated NSCLC patients harboring double mutations, because of the low incidence. Some articles have focused on patients with double activating mutations of 19Del and L858R [3, 8, 9]. As far as we know, only few studies have focused on patients with a combination of activating and resistant mutations before EGFR-TKI treatment [10, 17]. However, the results of these studies were inconsistent, so the clinical characteristics and response to EGFR-TKIs of those patients with double mutations remained controversial [3, 8–10, 17, 27–30]. The coexistence of activating EGFR mutation and pretreatment T790M mutation has been underestimated, despite accumulating evidence that the pretreatment T790M mutation occurs in approximately 35–60% of patients with EGFR-mutant NSCLC, depending on the detection method [31]. In the current study, we retrospectively explored the clinical characteristics and outcomes of EGFR-TKI therapy in patients with double activating mutations or a combination of activating and resistant mutations.

In previous studies of Asian patients, the frequency of double mutations 19Del + L858R ranged from 0.12% to 3.6% [3, 8, 9, 28, 32, 33]. The rate of patients harboring a combination of activating and resistant mutations, including 19Del + T790M and L858R + T790M, ranged from 0.06% to 1.6% [10, 17, 28, 32–34]. In the present study, the frequency of patients with double activating mutations and those with a combination of activating and resistant mutations was 0.67% (17/2546) and 1.1% (28/2546), respectively. In our study, patients with EGFR double mutations were more likely to be nonsmokers, have PS 0-1, have adenocarcinoma, and be at stage III-IV. These data were similar to previous data in an Asian population [32, 33]. As a new discovery, we found that patients with the double mutation 19Del + L858R were more likely to be male. Further, these double mutations were not so infrequent; thus the efficacy of EGFR-TKIs for patients with double mutations should be elucidated.

In the study of Zhang et al., 3 of 5 patients with EGFR double mutations of 19Del and L858R were treated with gefitinib. Of the 3 patients, 1 achieved a CR and 1 PR, and their PFS was 19 and 21 months, respectively. Further analysis demonstrated that cell lines with the double mutation of 19Del and L858R responded better than those with a single mutation when treated with EGFR-TKIs [3]. However, in the research of Wei et al., 21 patients with EGFR double mutations of 19Del and L858R received EGFR-TKIs. The ORR was only 23.8%, which was much lower than patients with a single EGFR-activating mutation [9]. Our study enrolled 11 patients with double mutations of 19Del and L858R treated with TKIs. The ORR in these patients was only 18.2%, which was also much lower than patients with single EGFR exon 19 or exon 21 mutations and was similar to the results of Wei et al. In our study, the ORR in patients with concomitant mutations of 19Del and T790M treated with TKIs was 50%, which was obviously higher than patients with 19Del + L858R or L858R + T790M concomitant mutations.

In the research of Wei et al. and Hata et al., the median PFS were 7.3 months and 16.5 months for patients with 19Del + L858R mutations, respectively [5, 9]. However, the median PFS for patients with 19Del + L858R was only 3.3 months in our study. Two possible explanations were speculated. Firstly, these differences might due to the heterogeneity of tumors. With small biopsy samples, tissue heterogeneity might lead to the analyzed sample being nonrepresentative of the tumor, causing misinterpretation of the mutation results [6, 7]. In addition, the small number of patients in all these studies was also a possible cause for these differences. Therefore, it may be difficult to draw a conclusion from these data. Future clinical trials with large numbers of patients are needed to provide a definitive conclusion.

The efficacy of TKI treatment for the patients with EGFR T790M mutation and one activating mutation remains controversial. Li et al. showed that the patients with EGFR T790M mutation and one activating mutation had significantly shorter OS than patients with a single activating mutation [10]. However, another study comparing erlotinib with chemotherapy as a first-line therapy in patients with EGFR mutations showed that patients harboring both an activating EGFR mutation and the T790M mutation had the best PFS outcome when treated with erlotinib, and survival was also longer in those patients [35]. Additionally, for patients with EGFR mutation treated with erlotinib and bevacizumab in the BELIEF study, the median progression-free survival was 16.0 months for those with T790M and 10.5 months for those without T790M (*P* = 0.016) [31]. Furthermore, compared with patients with L858R + T790M mutations or 19Del + L858R mutations, the PFS-TKI was also significantly longer for those patients with double mutations of 19Del and T790M in our study.

Although the mechanisms for the better response to TKIs in patients with 19Del + T790M compared to patients with double activating mutations (19Del + L858R) remain unclear, there are several hypotheses worthy of consideration. Firstly, perhaps the molecular conformation change of the EGFR tyrosine kinase domain induced by EGFR double mutations leads to the unexpected outcome. Compared with crystal structure changes made by a combination of 19Del and T790M mutations, coexistence of double activating mutations (19Del + L858R) might change the crystal structures and molecular conformation of the EGFR-TK adenosine triphosphate (ATP) binding site, resulting in decreased affinity for EGFR-TKIs [4, 19, 20, 36–38]. Secondly, the configuration of double mutations might affect how the cells respond to therapy. Three different scenarios could be speculated: (1) activating and resistant mutation in cis on the same allele, (2) in trans on different alleles, and (3) in different clones. In the first situation, cells were refractory to EGFR-TKIs. In the second scenario, tumor behavior would be difficult to anticipate, depending on which allele was expressed within the cells. The last situation might explain the favorable prognosis of T790M mutation, because clones with T790M mutation alone had been proven to be less oncogenic both in vitro and in vivo [39, 40]. Thirdly, in the presence of EGFR-activating mutations, NSCLC patients with a smoking history demonstrated poor response to EGFR-TKI treatment [41–43]. In our study, patients with double mutations of 19Del + L858R had a higher rate of smoking (63.6% versus 0% versus 20%, *P* = 0.0114) than the other 2 groups. This might be another reason why the patients in the 19Del + T790M group derived more benefit from EGFR-TKI treatment than patients with double mutations of 19Del + L858R. Fourthly, the differences in outcomes of EGFR-TKIs therapy are not only closely related to EGFR mutation types, but also other members of the signaling pathway, which may influence efficacy outcomes. Kim et al. [44] found that mutations of EGFR downstream genes are also closely related to the efficacy of TKIs. Finally, the heterogeneity of tumors might be another reason for inferior responses to EGFR-TKIs in patients with EGFR double activating mutation. Many experts have found that there are both mutated and wild-type cells within the same tumor. The temporal and spatial heterogeneity of tumors indicate that cancers are highly dynamic ecosystems. The fitter treatment-resistant cells would proliferate at the expense of the less fit treatment-sensitive cells with the progress of treatment [45–48]. Thus, the number of treatment-resistant cells might be more important for predicting ORR and PFS than the types of mutation.

Previous studies compared the clinical characteristics and response to TKIs between patients with 19Del mutation and patients with L858R mutation. EGFR-TKI treatment was more effective than chemotherapy for patients with 19Del mutation. However, for patients with L858R mutation, EGFR-TKI treatment and chemotherapy have similar effect and chemotherapy might even be better [49–51]. Interestingly, in our study, a short PFS was always appreciable if patients were carrying L858R mutation in combination with other mutation. In addition, previous studies also showed that L858R mutation in combination with other mutation exhibited a significant increase in phosphorylation and attenuated response to EGFR-TKI [9, 27, 35]. It indicated that outcomes of patients with L858R mutation should be redefined. Further studies were needed to confirm this finding.

There were some limitations in this study. First, the major limitation of this report was its retrospective nature, which might introduce potential bias resulting from uncontrolled factors involved in the complex treatment regimens, such as treatment duration and concurrent therapy, since NSCLC patients received a wide variety of treatments. Second, the number of patients with EGFR double mutations in this study was still not enough and might be insufficient to clearly draw a conclusion. Third, not all the patients in this study received periodic chest and brain imaging scans. Therefore, the timing and incidence of disease progression might be inaccurate. Finally, this study did not evaluate the clinical characteristics and outcomes of EGFR-TKI therapy in patients with EGFR double mutations, including rare EGFR mutations.

In summary, to our knowledge, our study is a retrospective clinical study describing the clinical characteristics and EGFR-TKI treatment outcomes of NSCLC patients with EGFR common double mutations, including 19Del, L858R, and T790M. We found that patients with common EGFR double mutations had a lower response to EGFR-TKI treatment than patients with a single activating mutation. In addition, patients with the double mutations of 19Del + T790M had higher ORR and longer PFS-TKI than patients with 19Del + L858R mutations or L858R + T790M mutations. The result of this study will help replenish EGFR double mutation data and the development of treatment strategies.

## Figures and Tables

**Table 1:** Baseline characteristics of 45 patients with EGFR double mutations.

Characteristics	Total *N* = 45 *n* (%)	19Del + L858R *N* = 17 *n* (%)	19Del + T790M *N* = 14 *n* (%)	L858R + T790M *N* = 14 *n* (%)	*X* ^2^	*P*
Gender					8.46	0.0145
Male	22 (48.9%)	13 (76.5%)	5 (35.7%)	4 (28.6%)		
Female	23 (51.1%)	4 (23.5%)	9 (64.3%)	10 (71.4%)		
Age (years)					3.23	0.1986
<60	26 (57.8%)	7 (41.2%)	10 (71.4%)	9 (64.3%)		
≥60	19 (42.2%)	10 (58.8%)	4 (28.6%)	5 (35.7%)		
Smoking					2.70	0.2588
Yes	13 (28.9%)	7 (41.2%)	2 (14.3%)	4 (28.6%)		
No	32 (71.1%)	10 (58.8%)	12 (85.7%)	10 (71.4%)		
Clinical stage					2.38	0.3048
I-II	13 (28.9%)	3 (17.6%)	6 (42.9%)	4 (28.6%)		
III-IV	32 (71.1%)	14 (82.4%)	8 (57.1%)	10 (71.4%)		
ECOG PS					1.18	0.5539
0-1	40 (88.9%)	14 (82.4%)	13 (92.9%)	13 (92.9%)		
2	5 (11.1%)	3 (17.6%)	1 (7.1%)	1 (7.1%)		
EGFR-TKIs					2.71	0.2578
Yes	24 (53.3%)	11 (64.7%)	8 (57.1%)	5 (35.7%)		
No	21 (46.7%)	6 (35.3%)	6 (42.9%)	9 (64.3%)		
Pathology					0.97	0.6145
Adenocarcinoma	43 (95.6%)	16 (94.1%)	13 (92.9%)	14 (100%)		
Squamous carcinoma	2 (4.4%)	1 (5.9%)	1 (7.1%)	0 (0%)		

**Table 2:** Baseline characteristics of 24 patients with EGFR double mutations who received EGFR-TKI therapy.

Characteristics	Total *N* = 24 *n* (%)	19Del + L858R *N* = 11 *n* (%)	19Del + T790M *N* = 8 *n* (%)	L858R + T790M *N* = 5 *n* (%)
Age (years)				
<60	14 (58.3%)	5 (45.5%)	6 (75%)	3 (60%)
≥60	10 (41.7%)	6 (54.5%)	2 (25%)	2 (40%)
Smoking				
Yes	8 (33.3%)	7 (63.6%)	0 (0%)	1 (20%)
No	16 (66.7%)	4 (36.4%)	8 (100%)	4 (80%)
ECOG PS				
0-1	20 (83.3%)	8 (72.7%)	7 (87.5%)	5 (100%)
2	4 (16.7%)	3 (27.3%)	1 (12.5%)	0 (0%)
Gender				
Male	13 (54.2%)	9 (81.8%)	3 (37.5%)	1 (20%)
Female	11 (45.8%)	2 (18.2%)	5 (62.5%)	4 (80%)
EGFR-TKI line				
First-line	10 (41.7%)	6 (54.5%)	2 (25%)	2 (40%)
After first-line	14 (58.3%)	5 (45.5%)	6 (75%)	3 (60%)
Pathology				
Adenocarcinoma	23 (95.8%)	11 (100%)	7 (87.5%)	5 (100%)
Squamous carcinoma	1 (4.2%)	0 (0%)	1 (12.5%)	0 (0%)

**Table 3:** Clinical variables and EGFR mutations associated with progression-free survival: multivariate analysis.

Variable	HR (hazard ratio)	95% CI	*P*
Mutation types (19Del + L858R/L858R + T790M)	0.422	0.082–2.182	0.303
Mutation types (19Del + T790M/L858R + T790M)	0.146	0.028–0.759	0.022
Mutation types (19Del + L858R/19Del + T790M)	0.347	0.070–1.719	0.195
Smoking (smoking/no smoking)	1.658	0.394–6.988	0.491
ECOG PS (0-1/2)	1.866	0.531–6.556	0.330
Gender (male/female)	1.015	0.275–3.749	0.982

## Data Access

All relevant data are within the paper and its Supporting Information files.

## Supplementary Materials

Supplementary Materials Table S1: baseline characteristics of patients with 19del + L858R versus single common EGFR mutation. Table S2: baseline characteristics of patients with 19Del + T790M versus single common EGFR mutation. Table S3: baseline characteristics of patients with L858R + T790M versus single common EGFR mutation. Table S4: the detail of patients with exon 19 deletion.
