# Supplementary material for: Clinical Characteristics and Survival Outcomes for Non-Small-Cell Lung Cancer Patients with Epidermal Growth Factor Receptor Double Mutations
Source: Biomed Res Int. 2018 Jan 16;2018:7181368. doi: 10.1155/2018/7181368 (PMC5822926; doi:10.1155/2018/7181368)
Supplement: Supplementary Materials — Table S1: baseline characteristics of patients with 19del + L858R versus single common EGFR mutation. Table S2: baseline characteristics of patients with 19Del + T790M versus single common EGFR mutation. Table S3: baseline characteristics of patients with L858R + T790M versus single common EGFR mutation. Table S4: the detail of patients with exon 19 deletion. [file 7181368.f1.pdf]

Table S1: Baseline characteristics of patients with 19del + L858R vs. single common EGFR mutation

Characteristics	19Del+L858R<br>*N*=17<br>*n* (%)	Single Common mutation<br>N=1126<br>*n* (%)	X^2	*P*
Gender			8.52	0.0035
Male	13(76.5%)	465(41.3%)		
Female	4(23.5%)	661(58.7%)		
Age (years)			1.81	0.1780
<60	7(41.2%)	647(57.5%)		
≥60	10(58.8%)	479(42.5%)		
Smoking			2.43	0.1189
Yes	7(41.2%)	278(24.7%)		
No	10(58.8%)	848(75.3%)		
Clinical stage			0.10	0.7461
I-II	3(17.6%)	167(14.8%)		
III-IV	14(82.4%)	959(85.2%)		
ECOG PS			0.80	0.3717
0-1	14(82.4%)	1004(89.2%)		
2	3(17.6%)	122(10.8%)		
Pathology			0.93	0.3356
Adenocarcinoma	16(94.1%)	1100(97.7%)		
Squamous carcinoma	1(5.9%)	26(2.3%)		

Table S2: Baseline characteristics of patients with 19Del+T790M vs. single common EGFR mutation

Characteristics	19Del+T790M<br>*N*=14<br>*n* (%)	Single Common mutation<br>N=1126<br>*n* (%)	X^2	*P*
Gender			0.18	0.6732
Male	5(35.7%)	465(41.3%)		
Female	9(64.3%)	661(58.7%)		
Age (years)			1.10	0.2932
<60	10(71.4%)	647(57.5%)		
≥60	4(28.6%)	479(42.5%)		
Smoking			0.81	0.3688
Yes	2(14.3%)	278(24.7%)		
No	12(85.7%)	848(75.3%)		
Clinical stage			8.44	0.0037
I-II	6(42.9%)	167(14.8%)		
III-IV	8(57.1%)	959(85.2%)		
ECOG PS			0.20	0.6581
0-1	13(92.9%)	1004(89.2%)		
2	1(7.1%)	122(10.8%)		
Pathology			1.40	0.2372
Adenocarcinoma	13(92.9%)	1100(97.7%)		
Squamous carcinoma	1(7.1%)	26(2.3%)		

Table S3: Baseline characteristics of patients with L858R+T790M vs. single common EGFR mutation

Characteristics	L858R+T790M N=14 n (%)	Single Common mutation N=1126 n (%)	X^2	P
Gender			0.92	0.3362
Male	4(28.6%)	465(41.3%)		
Female	10(71.4%)	661(58.7%)		
Age (years)			0.26	0.6076
<60	9(64.3%)	647(57.5%)		
≥60	5(35.7%)	479(42.5%)		
Smoking			0.11	0.7379
Yes	4(28.6%)	278(24.7%)		
No	10(71.4%)	848(75.3%)		
Clinical stage			2.05	0.1525
I-II	4(28.6%)	167(14.8%)		
III-IV	10(71.4%)	959(85.2%)		
ECOG PS			0.20	0.6581
0-1	13(92.9%)	1004(89.2%)		
2	1(7.1%)	122(10.8%)		
Pathology			0.33	0.5652
Adenocarcinoma	14(100%)	1100(97.7%)		
Squamous carcinoma	0(0%)	26(2.3%)		

The detail of patients with exon 19 deletion

Gender	Type of mutation	Type of gene change in 19Del	Smoking	Pathology	ECOG PS	Efficacy of EGFR-TKIs	Progression-free survival(month)
Male	19Del+L858R	2235-2249del15 (E746-A750del)	Yes	Adenocarcinoma	0-1	Stable Disease	15
Female	19Del+L858R	2235-2249del15 (E746-A750del)	No	Adenocarcinoma	0-1	Partial Response	11
Male	19Del+L858R	2236-2253del15 (E746-T751del)	No	Adenocarcinoma	0-1	Progressive Disease	-
Male	19Del+L858R	2239-2253del15 (L747-T751del)	Yes	Adenocarcinoma	0-1	Partial Response	8
Female	19Del+T790M	2235-2249del15 (E746-A750del)	No	Adenocarcinoma	0-1	Partial Response	21
Female	19Del+T790M	2240-2257del18 (L747-P753>S)	No	Adenocarcinoma	0-1	-	-
